# The Influence of Primary Care Quality on Hospital Admissions for People with Dementia in England: A Regression Analysis

**DOI:** 10.1371/journal.pone.0121506

**Published:** 2015-03-27

**Authors:** Panagiotis Kasteridis, Anne R. Mason, Maria K. Goddard, Rowena Jacobs, Rita Santos, Gerard McGonigal

**Affiliations:** 1 Centre for Health Economics, University of York, York, United Kingdom; 2 Department of Medicine for the Elderly, York Teaching Hospital NHS Foundation Trust, York, United Kingdom; Cardiff University, UNITED KINGDOM

## Abstract

**Objectives:**

To test the impact of a UK pay-for-performance indicator, the Quality and Outcomes Framework (QOF) dementia review, on three types of hospital admission for people with dementia: emergency admissions where dementia was the primary diagnosis; emergency admissions for ambulatory care sensitive conditions (ACSCs); and elective admissions for cataract, hip replacement, hernia, prostate disease, or hearing loss.

**Methods:**

Count data regression analyses of hospital admissions from 8,304 English general practices from 2006/7 to 2010/11. We identified relevant admissions from national Hospital Episode Statistics and aggregated them to practice level. We merged these with practice-level data on the QOF dementia review. In the base case, the exposure measure was the reported QOF register. As dementia is commonly under-diagnosed, we tested a predicted practice register based on consensus estimates. We adjusted for practice characteristics including measures of deprivation and uptake of a social benefit to purchase care services (Attendance Allowance).

**Results:**

In the base case analysis, higher QOF achievement had no significant effect on any type of hospital admission. However, when the predicted register was used to account for under-diagnosis, a one-percentage point improvement in QOF achievement was associated with a small reduction in emergency admissions for both dementia (-0.1%; P=0.011) and ACSCs (-0.1%; P=0.001). In areas of greater deprivation, uptake of Attendance Allowance was consistently associated with significantly lower emergency admissions. In all analyses, practices with a higher proportion of nursing home patients had significantly lower admission rates for elective and emergency care.

**Conclusion:**

In one of three analyses at practice level, the QOF review for dementia was associated with a small but significant reduction in unplanned hospital admissions. Given the rising prevalence of dementia, increasing pressures on acute hospital beds and poor outcomes associated with hospital stays for this patient group, this small change may be clinically and economically relevant.

## Introduction

The World Health Organisation [1] has declared dementia to be the leading cause of dependency and disability amongst older people in high, middle and lower income countries: an estimated 35.6 million people worldwide are living with dementia. With an estimated annual global cost to society of US$604 billion, it has been noted that, “if dementia care were a country, it would be the world’s 21st largest economy, ranking between Poland and Saudi Arabia” (ibid., p. 28). The number of people with dementia is predicted to double every 20 years (ibid., p. 90), trends that are echoed in England where dementia has been described by the British Prime Minister as “one of the biggest challenges we face today” [2] and is a top priority for action [3, 4]. At any one time, a quarter of acute hospital beds in England are in use by people with dementia, mainly due to falls (14%), hip fractures (12%), urinary tract infections (9%), chest infections (7%) and stroke (7%) [5]. Compared to people with the same underlying conditions but without dementia, hospital stays are longer and costs are higher [6, 7]. Entry into emergency care is a defining moment in the life of someone with dementia and often heralds an avoidable downward health spiral.

Despite some improvements, care for dementia remains poor and fragmented [4, 8–10]. Poor co-ordination, especially between health and social care, can lead to cost shifting and ‘problem dumping’—a major cause of poor care and inefficiency [11]. Lack of co-ordination between primary, secondary and social care is a key factor in triggering avoidable admissions, for elderly patients and specifically for those with dementia.

Since 2006/7, primary care practices in the UK have been paid to identify patients with dementia and to provide an annual review as part of the Quality and Outcomes Framework (QOF). The QOF is a pay-for-performance scheme that operates in all four countries of the UK. Primary care practices are rewarded for achieving clinical and organisational targets and on measures of self-reported patient experience. The annual review for dementia can be conceived as a broad measure of primary care quality. In the review, communication and coordination arrangements across care boundaries should be assessed; the support needs of the patient and their carer addressed; and the clinician should check the patient’s mental and physical health. Compared with their peers, people with dementia are at higher risk of depression and are less likely to report physical conditions [12]. Therefore, the review should increase the level of primary (ambulatory) care, outpatient and planned (elective) inpatient care. Insofar as it has a preventative effect, the health check may also reduce the rate of unplanned (emergency) hospital admissions [9, 13].

Given the substantial disease burden and financial costs associated with dementia, the identification of effective mechanisms for reducing avoidable emergency care and improving the detection of treatable conditions is a high national priority [14]. The primary objective of this study was to test whether the quality of primary care, as measured by the QOF annual dementia review, has an impact on hospital admissions for people with dementia in England.

## Methods

The analyses drew on data from national routine administrative datasets, and the unit of analysis was the primary care practice. We ran a series of count data regression models to test for an association between the quality of primary care, proxied by the QOF dementia review, and hospital admissions (Table 1). Our three research questions asked whether the QOF dementia review was associated with:

1. lower emergency admissions for dementia
2. lower emergency admissions for ambulatory care sensitive conditions (ACSCs) [15]
3. higher elective admissions for cataract, hip replacement, hernia, prostate disease, or hearing loss (conditions that might reasonably be identified at the annual QOF review).

**Table 1:** Overview of the 3 models in the GP level analyses.

**Model**	**Exposure**	**QOF dementia review indicator**	
		Achievement rate	Exception rate
Model BC: Base case	Dementia register	A/ [D+E]	E/ [D+E]
Model SA1: Sensitivity-1	Predicted register	A/ [D+E]	E/ [D+E]
Model SA2: Sensitivity-2	Dementia register	A/ D	E/ [D+E]

Legend: QOF = Quality and Outcomes Framework; A = QOF achievement (numerator); D = QOF denominator; E = patients exception-reported

Table 2 provides descriptive statistics for the variables used in the analyses.

**Table 2:** Descriptive statistics for regression samples.

Practice variable		mean	SD	min	max
*Dependent variables*	Admissions (emergency): dementia	1.0	1.3	0	27
	Admissions (emergency): ACSCs	3.0	3.0	0	42
	Admissions (elective)	0.3	0.6	0	6
*Exposure measures*	Dementia register (no. patients)	29.4	27.2	1	509
	Predicted register ^b^(no. patients)	73.7	68.2	1	816
*Quality (QOF) variables*	QOF achievement(%) ^a^	75.6	15.9	0	100
	Underlying QOF achievement (%) ^c^	81.9	14.6	0	100
	QOF Exception rate (%)	7.8	10.2	0	100
*GP practice characteristics* ^*a*^	No. patients on practice list (‘000s)	6.72	4.04	1	41
	% female GPs in practice	39.03	27.17	0	100
	Average age of GPs in practice (years)	48.00	7.63	28	76
	% UK qualified GPs in practice	67.34	37.55	0	100
	Single handed practice	0.16	0.36	0	1
	PMS practice	0.42	0.49	0	1
	Average age of practice patients (years)	38.97	4.11	22	56
	% male patients in practice	50.25	2.36	38	80
	% practice patients residing in urban areas	82.18	31.03	0	100
	% non-white patients in practice	11.27	15.31	0	81
	% 60+ in income deprivation in practice area	22.47	10.82	4	80
	% informal carers in practice area	9.90	1.34	5	15
*Access to care* ^*a*^	% report access to primary care within 48hrs	83.83	10.96	0	100
	Minimum distance from acute hospital (km)	3.85	3.81	0	36
*Social support* ^*a*^	% AA claimants	15.91	3.36	4	31
	% AA claimants paid higher rate	53.89	6.03	29	76
	% practice patients 65+ in nursing home	3.36	4.16	0	74

Legend: AA = attendance allowance; ACSC = ambulatory care sensitive condition; GP = general medical practitioner; N = number of observations; PMS = Personal Medical Services; QOF = Quality and Outcomes Framework; SD = standard deviation.

^a^ Values in Model BC (base case analysis. N = 39,362)

^b^ Model SA1 = Sensitivity-1 (N = 39,362)

^c^ Model SA2 = Sensitivity-2 (N = 39,335)

### Hospital admissions

To identify relevant admissions for our dependent variables, we used a four step approach. First, we mapped Read codes—the clinical codes used in UK primary care—for the QOF dementia register to the diagnostic codes used for hospital care (ICD10). This ensured that the criteria for identifying hospital admissions matched the eligibility criteria for the QOF dementia register. The Health and Social Care Information Centre (HSCIC) provided a mapping algorithm, and we checked this against a mapping based on a large UK primary care database, the Clinical Practice Research Datalink. The final list of ICD10 codes for dementia was: F00-F03, G300-G309, G310, G311, G318, F051 and F107. Second, we identified individuals in the Hospital Episode Statistics (HES) who had at least one of these codes as their primary or secondary diagnosis during the sample period 2006/07–2010/11. Third, we extracted three samples from HES based on three types of admission (i.e. our dependent variables). Fourth, we dropped all admissions that occurred before a patient’s first (inpatient) dementia diagnosis, and, to ensure consistency with QOF registers which are defined at the end of the financial year, admissions occurring during the year the patient died.

Emergency dementia admissions were defined as those where the patient had a primary diagnosis of dementia on admission. Emergency ambulatory care sensitive conditions were defined by specific ICD10 codes [15]. Elective conditions comprised cataract, hip replacement, hernia, prostate disease, or hearing loss, selected in consultation with our lay project advisors as those that might reasonably be identified at the QOF annual dementia review. For each admission type, we defined the dependent variable as the number of practice patients aged 18 or over with at least one admission to hospital within a year. Table 1 shows the three models that were tested with each of the three dependent variables (admission types), and Table 2 provides descriptive statistics.

### Quality of care

The primary explanatory variable of interest was the quality of care provided by the practice, which was proxied by the QOF indicator scores for the dementia review. The quality indicators for dementia were introduced in April 2006, and we compiled a set of panel data covering the financial years 2006/7 to 2010/11. QOF indicator scores are freely available at practice level (http://qof.hscic.gov.uk/), but are not published at patient level. We measured achievement based on the total number of people in the practice for whom the indicator was achieved, divided by the total number of eligible people (Table 1). We did not use QOF points or thresholds in the achievement measure, as points-based measures understate between-practice variation in quality and may be subject to gaming behaviour [16]. GPs may ‘exception report’ individuals who are considered unsuitable for treatment, or who are newly registered with the practice or newly diagnosed, or who make an informed dissent. In the base case analysis, we included exception-reported individuals in the denominator for the achievement calculation. A typical practice excludes (exception reports) around 8% of eligible patients from the dementia annual review [17], but clearly these individuals are still at risk of hospitalisation. To test the impact of exception reporting, the exception rate was included as a separate covariate in all analyses (i.e. additional to the achievement measure). As a sensitivity analysis, we also tested a modified quality indicator that excluded exception-reported patients from the denominator (see Table 1; Model SA2); this higher QOF achievement rate is the measure used for reimbursing practice performance. The average annual review rates were relatively stable over our sample period, but performance was sufficiently variable to allow differences within and between practices to be tested.

### Exposure terms

**Fig 1:**
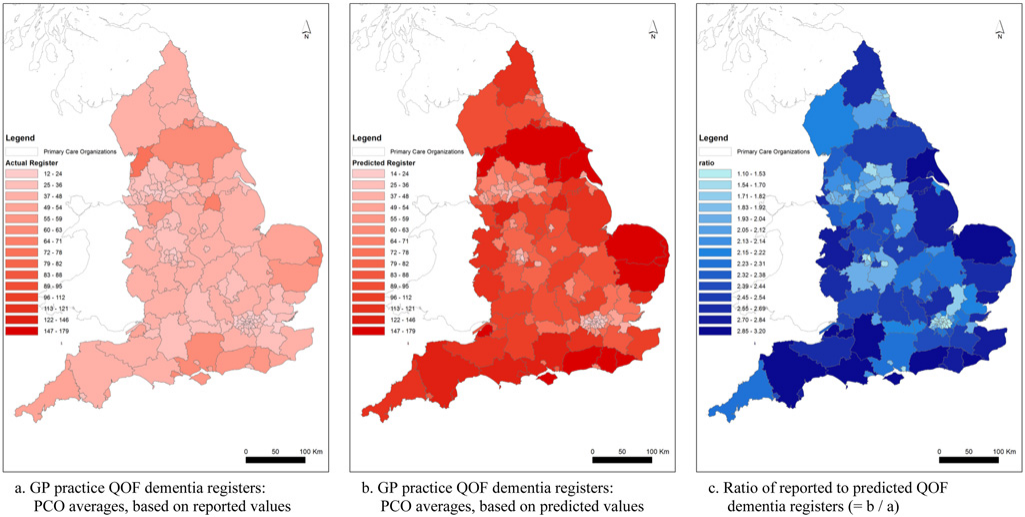
GP practice QOF dementia registers: average for English Local Health Authorities, 2010/11.

Our base case exposure term—the pool of individuals in a practice who were at risk of admission—was the reported QOF dementia register. However, less than half of those in England with dementia have a formal diagnosis [18], and the QOF dementia register is therefore highly likely to underestimate the ‘true’ disease prevalence. To test the robustness of findings, we used an alternative measure for our exposure term (Table 1; Model SA1). This alternative measure was derived from published estimates of ‘true’ population prevalence figures [19], officially considered to be “the most authoritative to date” [20]. We used age-gender band prevalence estimates for early and late onset dementia to predict the QOF register for each GP practice in each year, adjusting for the number of patients in nursing homes where prevalence is higher (details are provided in S1 Appendix). We tested the effect of this revised exposure term in our regressions. On average, the predicted register is more than twice the reported QOF dementia register (Table 2). In Fig. 1, the impact of this difference between the registers is mapped at local health authority (PCO, primary care organisation) level. The figure shows how PCO average register estimates vary when based on (a) the reported QOF dementia register and (b) the predicted register, with darker red indicating larger numbers. In map (c), darker blue indicates a larger change when moving from (a) to (b). These figures were generated using ArcGIS software and we used the same intervals in figures (a) and (b) to facilitate comparison.

### Other covariates

Hospital admissions are influenced by factors other than the quality of primary care, and people with dementia often have complex health and social care needs. Our analyses therefore adjusted for an array of factors (Table 2). These were selected in consultation with lay advisors to the research project, who have experience of caring for family members with dementia.

To control for primary care practice characteristics, we used data from the General Medical Services (GMS) dataset, QOF dataset and Attribution Dataset (ADS). To control for local population characteristics, we used data from the ONS Neighbourhood Statistics which are available at Lower Super Output Area (LSOA) level. LSOAs are defined geographic units that cover an average population of 1,500 people. As defined by the 2001 Census, there were 32,482 LSOAs in England during the study period (2006/7 to 2010/11). The ADS provides a breakdown of the practice population by LSOA. We derived a weighted average of the local population characteristics of the practice (e.g. deprivation, ethnicity, informal care) and assigned this to the practice. ADS data are collected at the start of the financial year, whereas QOF data are collected at the end of the financial year. We therefore adjusted the estimates based on ADS by taking moving averages across two years of data.

We derived two measures to reflect differences between practices in terms of access to care. First, the variable on 48-hour access to primary care came from the annual GP Patient Survey. Second, to adjust for patients’ geographical access to emergency care we estimated the straight line distance from the primary care practice to the nearest acute hospital, using postcode data for primary care practices and acute hospitals from the HSCIC. Calculations were based on the grid reference from the postcodes of all the practice branches and of acute hospitals [21]. Straight line distances are a good proxy for road distances, and are less computationally demanding [22, 23].

The level of social support provided in the community may help prevent acute hospital admissions [24]. We used three measures of social support: the proportion of practice patients aged 65 and over who were nursing homes residents, based on time series data from the HSCIC; and two variables based on Attendance Allowance (AA) data from the Department of Work and Pensions. AA is a social benefit for people aged 65 or over who have a physical or mental disability and need help with personal care. It is not means tested, is available at lower and higher rates depending on need, and can be used to pay for care from any provider. We derived two practice-level measures: the percentage of people claiming AA; and the percentage of AA claimants who were paid at the higher rate. Around 16 percent of practice patients aged 65 and over claim AA, with over half of those receiving the higher rate.

Our practice-level measure of deprivation was the percentage of people aged 60 or over living in income deprivation. Uptake of AA was positively correlated with this measure of deprivation, but the relationship was not linear. We therefore used a set of interaction terms to test whether the effect of AA varied depending on the level of deprivation. Specifically, we interacted the percentage of AA claimants in the practice with a categorical measure of deprivation that grouped practices according to whether the percentage of people aged 60 or over living in income deprivation was low (<20%; 50% of practices), medium (20% to 35%; 37% of practices) or high (> 35%; 13% of practices).

### Model specification and statistical analysis

After excluding patients younger than 18 and those who changed practices within a year, we merged the HES patient-level data with the QOF practice-level data and linked these to the covariates derived from the other datasets. We dropped practices with a list size of fewer than 1,000 patients because these practices have relatively small numbers of patients with dementia and practice performance is therefore subject to large fluctuations over time. Our final dataset provided an unbalanced panel of between 7,697 and 7,965 GP practices per year, with most practices (82.5%) contributing five years of data.

We ran panel count data models to test the impact of primary care quality on hospital admissions. Our response variable was defined as the number of people admitted to hospital *adm*_*it*_ at least once in year *t* = 2006/07,…,2010/11 from GP practice *i* = 1,…,*N*. Around 11% of practices had no emergency admissions for dementia over the 5 year study period, and for elective admissions the figure was over 40%. As these practices could not contribute data to fixed effects models, we estimated random effects Poisson models for the three admission types specified as follows:

$$adm_{it}∼P[\alpha_{i}*risk_{it}*\exp(Q_{it}\gamma +{X^{\prime }}_{it}\beta +D_{t}\lambda )]$$ (1)

In this equation, *Q*_*it*_ is the variable for GP practice quality as measured by the QOF; *X*_*it*_ is a vector of covariates that capture differences in the practice patient population and the supply of and access to other care resources; *D*_*t*_ is a vector of time dummy variables to control for temporal trends in hospital admission; *risk*_*it*_ is the number of patients at risk of admission which in our base case analysis (Model BC) is defined as the QOF dementia register (see subsection: Exposure terms).

The GP practice-specific effects *α*_*i*_ capture unobserved, time-invariant practice effects, in other words any practice behaviour that influences admission rates, including, but not limited to, decisions specifically on hospital care. Following the convention we assume a conjugate gamma density for the multiplicative GP practice-specific random effects [25].

Estimation of random effects is carried out by maximizing the likelihood that results after integrating out the conjugate random effects [26]. We calculated bootstrapped standard errors for the random effects model. Statistical significance was assessed at the 5%, 1% and 0.1% levels.

The coefficient estimates can be interpreted as the percentage change in admissions resulted from a *partial* change in a variable (semi-elasticity). The exponentiated coefficients or incidence rate ratios (IRRs) also facilitate a semi-elasticity interpretation with (IRR-1)*100 estimating the result of a *discrete* (one percentage point) change in the QOF rate on the percentage change in admissions. For instance, an IRR of 0.9975 implies a reduction in admissions of 0.25%. Details of the method used to calculate IRRs for the AA and deprivation variables which involve interaction terms are provided in S2 Appendix.

We ran two sensitivity analyses to test the robustness of findings (Table 1). First, we used an alternative measure for our exposure term (Model SA1; Sensitivity-1). Second, we tested a modified measure of achievement that excluded exception-reported patients (Model SA2; Sensitivity-2), as this measure is the basis on which practices are rewarded. Analyses were performed using Stata, version 12 (StataCorp LP, TX).

### Ethics statement

This was a retrospective analysis of previously collected, non-identifiable information, and involved no change in the management of patients. Obtaining individual consent was not feasible so patient records were anonymized and de-identified prior to analysis. The Health and Social Care Information Centre (HSCIC) handles requests for de-identified data and has a legal responsibility to ensure there is an appropriate legal basis to permit the release and subsequent processing of data, that all necessary approvals are in place, and that organisations have appropriate arrangements and safeguards for secure data handling. The HSCIC approved the release of the Hospital Episode Statistics (http://www.hscic.gov.uk/hes) data to the University of York (Data Re-Use Agreements RU115; RU536; RU750).

## Results

In the base case analysis, higher QOF achievement had no significant effect on any type of hospital admission (Table 3). When the predicted register was used to account for under-diagnosis (Model SA1), a one-percentage point improvement in QOF achievement was associated with a small but statistically significant reduction in emergency admissions for both dementia (-0.1%; P = 0.011) and ACSCs (-0.1%; P = 0.001) (Table 4).

**Table 3:** Effects of the QOF review on hospital admissions: results from the base case analysis (Model BC).

	Emergency admissions for dementia			Emergency admissions for ACSCs			Elective admissions		
	IRR	95% CI	P value	IRR	95% CI	P value	IRR	95% CI	P value
*QOF variables*									
QOF achievement rate	0.999	(0.998, 1.000)	0.080	1.000	(0.999, 1.000)	0.286	0.999	(0.997, 1.001)	0.426
QOF exception rate	1.002	(1.000, 1.004)	0.015	0.999	(0.998, 1.000)	0.117	0.998	(0.995, 1.001)	0.142
*GP practice characteristics*									
Mean age of practice pop	1.004	(0.998, 1.009)	0.231	0.994	(0.990, 0.998)	0.004	1.002	(0.993, 1.010)	0.662
% male patients	1.008	(1.001, 1.016)	0.029	0.998	(0.991, 1.004)	0.494	1.018	(1.002, 1.034)	0.028
% female GPs	1.000	(0.999, 1.000)	0.317	0.999	(0.999, 1.000)	0.000	1.000	(0.999, 1.001)	0.952
Mean age of GPs	1.004	(1.001, 1.007)	0.005	1.000	(0.999, 1.002)	0.704	1.006	(1.002, 1.010)	0.002
% UK qualified GPs	1.000	(0.999, 1.000)	0.202	0.998	(0.998, 0.999)	0.000	1.000	(0.999, 1.001)	0.850
PMS practice	0.975	(0.947, 1.003)	0.084	0.989	(0.972, 1.007)	0.227	1.043	(0.993, 1.095)	0.090
Single handed practice	1.000	(0.974, 1.093)	0.291	1.000	(0.991, 1.063)	0.146	1.000	(0.881, 1.120)	0.915
No. registered patients (practice list)	0.994	(0.989, 0.999)	0.009	0.993	(0.990, 0.996)	0.000	0.996	(0.991, 1.002)	0.175
% patients residing in urban areas	1.001	(1.000, 1.002)	0.002	1.002	(1.002, 1.003)	0.000	1.000	(0.999, 1.001)	0.917
% non-white patients	0.997	(0.995, 0.998)	0.000	1.003	(1.002, 1.004)	0.000	0.999	(0.997, 1.002)	0.492
% informal carers	1.007	(0.990, 1.024)	0.436	1.008	(0.995, 1.020)	0.216	0.997	(0.973, 1.020)	0.769
*Access to care*									
Minimum distance for acute hospital	0.995	(0.991, 1.000)	0.046	0.995	(0.992, 0.998)	0.004		
% report access within 48hrs	0.997	(0.996, 0.999)	0.000	0.997	(0.996, 0.998)	0.000	0.996	(0.994, 0.998)	0.000
% 60+ in low deprivation *[reference]*								
% 60+ in medium deprivation	1.310	(1.094, 1.569)	0.003	1.272	(1.125, 1.438)	0.000	1.002	(0.717, 1.401)	0.990
% 60+ in high deprivation	2.584	(1.858, 3.594)	0.000	1.846	(1.496, 2.277)	0.000	0.814	(0.453, 1.462)	0.492
*Social support*									
% AA claimants (low deprivation)	1.012	(1.002, 1.022)	0.017	1.006	(1.001, 1.012)	0.032	0.994	(0.982, 1.006)	0.330
% AA claimants (med. deprivation)	1.002	(0.993, 1.011)	0.693	1.000	(0.993, 1.006)	0.919	0.997	(0.981, 1.013)	0.732
% AA claimants (high deprivation)	0.976	(0.962, 0.991)	0.002	0.982	(0.973, 0.992)	0.000	1.007	(0.979, 1.035)	0.603
% AA claimants paid higher rate	1.011	(1.008, 1.014)	0.000	1.003	(1.002, 1.005)	0.000	1.003	(0.998, 1.007)	0.231
% patients 65+ in nursing home	0.954	(0.950, 0.959)	0.000	0.982	(0.978, 0.985)	0.000	0.958	(0.951, 0.965)	0.000
*Year dummies*									
Year 2006/07*[reference]*									
Year 2007/08	0.939	(0.909, 0.970)	0.000	1.111	(1.088, 1.135)	0.000	1.473	(1.354, 1.603)	0.000
Year 2008/09	0.867	(0.838, 0.898)	0.000	1.289	(1.261, 1.318)	0.000	1.762	(1.643, 1.890)	0.000
Year 2009/10	0.852	(0.824, 0.880)	0.000	1.390	(1.357, 1.424)	0.000	2.075	(1.941, 2.218)	0.000
Year 2010/11	0.729	(0.701, 0.758)	0.000	1.434	(1.396, 1.473)	0.000	2.351	(2.210, 2.501)	0.000

Legend: AA = attendance allowance; ACSC = ambulatory care sensitive condition; CI: confidence interval; GP = general medical practitioner; IRR = incidence rate ratio; QOF = Quality and Outcomes Framework (annual dementia review indictor); PMS = Personal Medical Services.

**Table 4:** Regression results for key variables: all models.

	**Emergency admissions for dementia**			**Emergency admissions for ACSCs**			**Elective admissions**		
	Incidence rate ratio (95% CI) [P Value]			Incidence rate ratio (95% CI) [P Value]			Incidence rate ratio (95% CI) [P Value]		
	*Model BC*	*Model SA1*	*Model SA2*	*Model BC*	*Model SA1*	*Model SA2*	*Model BC*	*Model SA1*	*Model SA2*
QOF achievement rate	0.999	0.999	0.999	1.000	0.999	1.000	0.999	0.999	0.999
	(0.998, 1.000)	(0.998, 1.000)	(0.998, 1.000)	(0.999, 1.000)	(0.999, 1.000)	(0.999, 1.000)	(0.997, 1.001)	(0.997, 1.001)	(0.998, 1.001)
	[0.080]	[0.011]	[0.068]	[0.286]	[0.001]	[0.464]	[0.426]	[0.297]	[0.525]
QOF exception rate	1.002	1.001	1.003	0.999	0.999	0.999	0.998	0.996	0.998
	(1.000, 1.004)	(0.999, 1.002)	(1.001, 1.005)	(0.998, 1.000)	(0.997, 1.000)	(0.998, 1.000)	(0.095, 1.001)	(0.093, 0.999)	(0.995, 1.001)
	[0.015]	[0.300]	[0.000]	[0.117]	[0.004]	[0.147]	[0.142]	[0.011]	[0.235]
% AA claimants (low deprivation)	1.012	1.015	1.012	1.006	1.010	1.006	0.994	0.998	0.994
	(1.002, 1.022)	(1.006, 1.024)	(1.003, 1.021)	(1.001, 1.012)	(1.004, 1.015)	(0.999, 1.013)	(0.982, 1.006)	(0.986, 1.010)	(0.980, 1.008)
	[0.017]	[0.001]	[0.008]	[0.032]	[0.001]	[0.076]	[0.330]	[0.744]	[0.375]
% AA claimants (med. deprivation)	1.002	0.989	1.002	1.000	0.987	1.000	0.997	0.983	0.997
	(0.993, 1.011)	(0.982, 0.997)	(0.991, 1.012)	(0.993, 1.006)	(0.980, 0.993)	(0.933, 1.006)	(0.981, 1.014)	(0.967, 0.999)	(0.981, 1.013)
	[0.693]	[0.006]	[0.737]	[0.919]	[0.000]	[0.933]	[0.732]	[0.040]	[0.721]
% AA claimants (high deprivation)	0.976	0.955	0.976	0.982	0.965	0.982	1.007	0.988	1.008
	(0.962, 0.991)	(0.940, 0.971)	(0.962, 0.990)	(0.973, 0.992)	(0.954, 0.975)	(0.972, 0.992)	(0.979, 1.035)	(0.959, 1.017)	(0.983, 1.034)
	[0.002]	[0.000]	[0.001]	[0.000]	[0.000]	[0.001]	[0.603]	[0.415]	[0.524]
% AA claimants paid higher rate	1.011	1.015	1.011	1.003	1.008	1.003	1.003	1.006	1.003
	(1.008, 1.014)	(1.013, 1.018)	(1.008, 1.014)	(1.002, 1.005)	(1.006, 1.009)	(1.001, 1.005)	(0.998, 1.007)	(1.002, 1.010)	(0.999, 1.007)
	[0.000]	[0.000]	[0.000]	[0.000]	[0.000]	[0.001]	[0.231]	[0.003]	[0.194]
% patients 65+ in nursing home	0.954	0.950	0.954	0.981	0.976	0.982	0.958	0.953	0.958
	(0.950, 0.959)	(0.944, 0.955)	(0.950, 0.959)	(0.978, 0.985)	(0.972, 0.980)	(0.979, 0.984)	(0.951, 0.965)	(0.946, 0.961)	(0.950, 0.966)
	[0.000]	[0.000]	[0.000]	[0.000]	[0.000]	[0.000]	[0.000]	[0.000]	[0.000]

Legend: Model BC = base case analysis; Model SA1 = Sensitivity-1 (using predicted register as exposure); Model SA2 = Sensitivity-2 (alternative QOF achievement measure)

AA = attendance allowance; ACSC = ambulatory care sensitive conditions; CI: confidence interval; QOF = Quality and Outcomes Framework (annual dementia review indictor).

In the sensitivity analysis where the QOF indicator used for reimbursement (A/D) was used in place of the base case QOF achievement measure (Model SA2), results did not differ from the base case.

The effects of QOF exception reporting were also sensitive to assumptions about under-diagnosis (Table 4). In the base case, a one percentage point increase in the QOF exception-reporting rate was associated with a small increase (0.2%) in emergency admissions for dementia (P = 0.015), but had no impact on emergency admissions for ACSCs or on elective admissions. When the predicted register was used (Model SA1), exception reporting had no significant effect on dementia admissions, but was associated with lower admissions for ACSC and lower elective admissions.

Practices with larger list sizes (more registered patients) had significantly fewer emergency admissions. With regard to access to care, practices with a higher percentage of patients able to make appointments within 48 hours had significantly fewer admissions of all three types. Greater distance to the nearest acute hospital was associated with lower emergency admissions for both dementia and ACSCs.

Compared to practices with low levels of income deprivation, practices with medium (high) levels of deprivation had 31% (258%) more emergency dementia admissions, but these large effects took no account of the interaction between deprivation and AA. As the proportion of AA claimants within a practice increased, the effects of deprivation diminished. At the mean value of AA uptake (15.9%), a high level of deprivation was associated with an increase in admissions of 11.7%. In practices where a smaller proportion of older people were living in income deprivation, higher levels of uptake of the social benefit were associated with more emergency admissions (Table 4). In practices with greater deprivation, the reverse effects were observed: emergency admissions were significantly lower, with a one percentage point increase in uptake of AA associated with a decrease in emergency admissions of -2.4% (dementia admissions) and -1.8% (ACSCs). In all analyses, practices with a higher proportion of nursing home patients had significantly fewer emergency admissions for dementia (ranging from -4.6% to -5.0%) and ACSCs (-1.8% to -2.4%) and significantly lower elective admissions (-4.2% to -4.7%) (P< 0.001).

## Discussion

Our study found that the annual health check for dementia undertaken by primary care practices as part of the QOF may be associated with reduced emergency hospital admissions in people with dementia. However, the impact was small and its statistical significance depended on the method for estimating disease prevalence. Given the rising prevalence of dementia, the increasing pressure on acute hospital beds and the poor outcomes associated with hospital stays for this patient group [9], this small change may, nonetheless, be clinically and economically relevant. It could also be cost-effective, although further research is needed to test this. Our study found no evidence of a link between annual health checks and elective admissions for specific conditions.

Some types of interventions may be more effective in reducing unplanned hospital admissions. A systematic review found lower rates of admission in practices with better preventative prescribing for asthma, and in practices with diabetes clinics; but asthma clinics and higher quality primary care for diabetes had no discernible impact [27]. Evidence on the impact of QOF performance on admissions is similarly mixed [28]. Negative associations (lower admissions) have been reported for diabetes [29, 30], angina [31], stroke [32] and epilepsy [33]; and no associations, or mixed effects, have been reported for asthma, chronic obstructive pulmonary disease and coronary heart disease [34–36].

Similar evidence in relation to dementia is scarce. A large study of Medicare claims data reported that those with a dementia diagnosis were more than three times as likely as others to have a hospital admission and more than twice as likely to have an ACSC hospital admission, pointing to potential failures in the ambulatory care sector [37]. An English study found that people with dementia were at higher risk of emergency admission [38]. Most of these admissions were for ACSCs, rather than dementia, and other studies confirm that ACSCs are prevalent in this inpatient group [39, 40].

This research adds to the sparse literature on the relationship between primary and secondary care for dementia patients and in particular on the ‘protective’ effect of higher quality primary care (as measured by the QOF) on emergency hospital admissions. Whilst this association does not necessarily imply the relationship is causal, we have made efforts to ensure the robustness of our methods and undertaken extensive sensitivity analyses to demonstrate our associations are qualitatively consistent. Our study quantifies the relationship using panel data to account for potential confounding effects, and is the first to explore the impact of under-diagnosis on the relationship between quality of primary care and hospital admissions. Another novel finding is that a social benefit, Attendance Allowance, was consistently associated with lower unplanned admissions (typically by around 2%) in practices where a higher proportion older people were living in income deprivation. Efforts to remove barriers to accessing social support could therefore be targeted towards these disadvantaged groups, and policy makers may also wish to investigate the level of benefit needed to prevent admissions. However, further research is needed to verify these findings and to guide policy.

There are some limitations of our study that could be addressed in future research. First, the QOF review targets the needs of both dementia patients and the support needs of their caregivers. The vital role that caregivers have in supporting people with dementia is reflected in the fact that two-thirds of dementia patients live at home, most receiving care from their family members [19]. Protecting caregivers’ health and wellbeing is considered to be a major factor in preventing crises and preventing or delaying admission to hospital or long-term residential care [41, 42]. However, the lack of individual-level data on caregivers meant that we could only adjust for the provision of informal care at the small area level.

Second, admissions were measured at patient level whilst QOF performance was at practice level. This meant it was unclear whether or when individuals who were admitted to hospital had received a QOF dementia review, or what other types of care they had received outside of hospital. A multilevel analysis using individual-level primary care data that takes account of the clustering of patients within practices and of admissions within hospitals would be needed to test the validity of our findings.

Third, future analyses could incorporate additional confounding factors. For instance, hospital admission may be influenced by the availability of nursing home beds, or intermediate care facilities. However, some important factors are difficult to capture in a statistical model, such as continuity of primary care, the quality of the doctor/patient interaction, the level of integration across health and social care settings, the expertise of individual practitioners, and access to services such as memory clinics or respite services for carers. Furthermore, routine data rarely include information on disease severity or frailty. More generally, dementia diagnoses are likely to be underreported, although the introduction of the dementia case-finding financial incentive scheme for English hospitals, FAIR (Find, Assess, Investigate and Refer), in 2012/13 should help address this particular issue. In addition, the use of routine administrative data always presents challenges in terms of data recording and accuracy.

Currently, around 35.6 million individuals world-wide are estimated to have dementia. That number is expected to double by 2030 and to triple by 2050 [1]. In the absence of clinically effective pharmacological interventions for dementia, ‘usual care’ will continue to be defined by a package of services designed to address the health and care needs of individuals with dementia and their caregivers. Further research on how to improve the quality and effectiveness of those services is therefore essential.

## Supporting Information

S1 Appendix Methodology for adjusting the predicted dementia registers (PDF)

S2 Appendix Calculation of IRR for variables involving interaction terms (PDF)

## References

[1] World Health Organization, Alzheimer's Disease International. Dementia: A Public Health Priority. Geneva: World Health Organization; 2012 10.1186/alzrt143

[2] Department of Health. The Prime Minister's Challenge on Dementia: delivering major improvements in dementia care and research by 2015. London: Department of Health; 2012.

[3] Department of Health. The Operating Framework for the NHS 2012/13. London: Department of Health; 2011.

[4] Department of Health. Living Well With Dementia: a national dementia strategy. London: Department of Health; 2009.

[5] Royal College of Psychiatrists, Centre for Quality Improvement (CCQI). Report of the National Audit of Dementia Care in General Hospitals 2012–13: second round audit report and update. Young J, Hood C, Gandesha A, editors. Royal College of Psychiatrists: London; 2013

[6] Lakey L. Counting the cost: caring for people with dementia on hospital wards. London: Alzheimer’s Society; 2009.

[7] Lin PJ, Fillit HM, Cohen JT, Neumann PJ. Potentially avoidable hospitalizations among Medicare beneficiaries with Alzheimer's disease and related disorders. Alzheimers Dement. 2013;9(1):30–8. 10.1016/j.jalz.2012.11.002 23305822

[8] Audit Commission. Forget me not: mental health services for older people National Report. London: Audit Commission; 2000.

[9] Care Quality Commission. The state of health care and adult social care in England in 2012/13. London: The Stationery Office; 2013.

[10] Care Services Improvement Partnership. Everybody's business: integrated mental health services for older adults: a service development guide. London: Department of Health; 2005.

[11] Knapp M, Iemmi V, Romeo R. Dementia care costs and outcomes: a systematic review. Int J Geriatr Psychiatry. 2013;28(6):551–61. 10.1002/gps.3864 22887331

[12] National Institute for Health and Care Excellence. Q30: Quality standard for supporting people to live well with dementia. 2013.

[13] Purdy S, Griffin T, Salisbury C, Sharp D. Ambulatory care sensitive conditions: terminology and disease coding need to be more specific to aid policy makers and clinicians. Public Health. 2009;123(2):169–73. 10.1016/j.puhe.2008.11.001 19144363

[14] NHS England Urgent and Emergency Care Review Team. Transforming urgent and emergency care services in England Update on the Urgent and Emergency Care Review. Leeds: NHS England; 2014.

[15] Bardsley M, Blunt I, Davies S, Dixon J. Is secondary preventive care improving? Observational study of 10-year trends in emergency admissions for conditions amenable to ambulatory care. BMJ Open. 2013;3(1):e002007 10.1136/bmjopen-2012-002007 23288268 PMC3549201

[16] Gravelle H, Sutton M, Ma A. Doctor behaviour under a pay for performance contract: treating, cheating and case finding? Econ J. 2010;120:F129–F56. 10.1111/j.1468-0297.2009.02340.x 21116477

[17] Doran T, Kontopantelis E, Fullwood C, Lester H, Valderas JM, Campbell S. Exempting dissenting patients from pay for performance schemes: retrospective analysis of exception reporting in the UK Quality and Outcomes Framework. Br Med J. 2012;344:e2405 10.1136/bmj.e2405 22511209 PMC3328418

[18] Department of Health. Dementia: A state of the nation report on dementia care and support in England. London: Department of Health; 2013.

[19] Knapp M, Prince M, Albanese E, Banerjee S, Dhanasiri S, Fernandez J-L, et al Dementia UK: a report into the prevalence and cost of dementia. London: Alzheimer’s Society, 2007.

[20] HSCIC Clinical Indicators Team. NHS Outcomes Framework 2014/15: Domain 2—Enhancing quality of life for people with long-term conditions Indicator specification. Health and Social Care Information Centre, 2014 Contract No.: Version 1.1.

[21] Nicholl J, West J, Goodacre S, Turner J. The relationship between distance to hospital and patient mortality in emergencies: an observational study. Emerg Med J. 2007;24(9):665–8. 17711952 10.1136/emj.2007.047654 PMC2464671

[22] Bliss RL, Katz JN, Wright EA, Losina E. Estimating proximity to care: are straight line and zipcode centroid distances acceptable proxy measures? Med Care. 2012;50(1):99–106. 10.1097/MLR.0b013e31822944d1 22167065 PMC3240808

[23] Dusheiko M, Halls P, Richards W. The effect of travel distance on patient non-attendance at hospital outpatient appointment: a comparison of straight line and road distance measures In: Fairbairn D, editor. GISRUK 2009: Proceedings of the GIS Research UK 17th Annual Conference; 2009 4 1–3; Durham, UK: University of Durham; 2009. p. 1–6.

[24] Forder J. Long-term care and hospital utilisation by older people: an analysis of substitution rates. Health Econ. 2009;18(11):1322–38. 10.1002/hec.1438 19206085

[25] Hausman J, Hall BH, Griliches Z. Econometric Models for Count Data with an Application to the Patents—R&D Relationship. Econometrica. 1984;52(4):909–38.

[26] Cameron AC, Trivedi PK. Regression analysis of count data Econometric Society Monographs, no. 30. Cambridge; New York and Melbourne: Cambridge University Press; 1998.

[27] Purdy S. Avoiding hospital admissions: What does the research evidence say? London: The King's Fund, 2010.

[28] Gillam SJ, Siriwardena AN, Steel N. Pay-for-performance in the United Kingdom: impact of the quality and outcomes framework: a systematic review. Ann Fam Med. 2012;10(5):461–8. 10.1370/afm.1377 22966110 PMC3438214

[29] Dusheiko M, Doran T, Gravelle H, Fullwood C, Roland M. Does higher quality of diabetes management in family practice reduce unplanned hospital admissions? Health Serv Res. 2011;46(1 Pt 1):27–46. 10.1111/j.1475-6773.2010.01184.x 20880046 PMC3034260

[30] Bottle A, Millett C, Xie Y, Saxena S, Wachter RM, Majeed A. Quality of primary care and hospital admissions for diabetes mellitus in England. J Ambul Care Manage. 2008;31(3):226–38. 10.1097/01.JAC.0000324668.83530.6d 18574381

[31] Purdy S, Griffin T, Salisbury C, Sharp D. Emergency admissions for coronary heart disease: A cross-sectional study of general practice, population and hospital factors in England. Public Health. 2011;125(1):46–54. 10.1016/j.puhe.2010.07.006 21183192

[32] Soljak M, Calderon-Larranaga A, Sharma P, Cecil E, Bell D, Abi-Aad G, et al Does higher quality primary health care reduce stroke admissions? A national cross-sectional study. Br J Gen Pract. 2011;61(593):e801–7. 10.3399/bjgp11X613142 22137417 PMC3223778

[33] Shohet C, Yelloly J, Bingham P, Lyratzopoulos G. The association between the quality of epilepsy management in primary care, general practice population deprivation status and epilepsy-related emergency hospitalisations. Seizure. 2007;16(4):351–5. 17395500 10.1016/j.seizure.2007.02.005

[34] Downing A, Rudge G, Cheng Y, Tu YK, Keen J, Gilthorpe MS. Do the UK government's new Quality and Outcomes Framework (QOF) scores adequately measure primary care performance? A cross-sectional survey of routine healthcare data. BMC Health Serv Res. 2007;7(1):166. 17941984 10.1186/1472-6963-7-166 PMC2117011

[35] Bottle A, Gnani S, Saxena S, Aylin P, Mainous AG, Majeed A. Association between quality of primary care and hospitalization for coronary heart disease in England: a national cross-sectional study. J Gen Intern Med. 2008;23(2):135–41. 17924171 10.1007/s11606-007-0390-2 PMC2359159

[36] Calderón-Larrañaga A, Carney L, Soljak M, Bottle A, Partridge M, Bell D, et al Association of population and primary healthcare factors with hospital admission rates for chronic obstructive pulmonary disease in England: national cross-sectional study. Thorax. 2011;66(3):191–6. 10.1136/thx.2010.147058 Erratum in Thorax. 2013 8;68(8):781. 21076143

[37] Bynum JPW, Rabins PV, Weller W, Niefeld M, Anderson GF, Wu AW. The relationship between a dementia diagnosis, chronic illness, medicare expenditures, and hospital use. J Am Geriatr Soc. 2004;52(2):187–94. 14728626 10.1111/j.1532-5415.2004.52054.x

[38] Natalwala A, Potluri R, Uppal H, Heun R. Reasons for hospital admissions in dementia patients in Birmingham, UK, during 2002–2007. Dement Geriatr Cogn Disord. 2008;26(6):499–505. 10.1159/000171044 19005254

[39] Sampson EL, Blanchard MR, Jones L, Tookman A, King M. Dementia in the acute hospital: prospective cohort study of prevalence and mortality. Br J Psychiatry. 2009;195(1):61–6. 10.1192/bjp.bp.108.055335 19567898

[40] Toot S, Devine M, Akporobaro A, Orrell M. Causes of Hospital Admission for People With Dementia: A Systematic Review and Meta-Analysis. J Am Med Dir Assoc. 2013;14(7):463–70. 10.1016/j.jamda.2013.01.011 23510826

[41] Banerjee S, Murray J, Foley B, Atkins L, Schneider J, Mann A. Predictors of institutionalisation in people with dementia. J Neurol Neurosurg Psychiatry. 2003;74(9):1315–6. 12933944 10.1136/jnnp.74.9.1315 PMC1738636

[42] Vroomen JM, Bosmans JE, van Hout HP, de Rooij SE. Reviewing the definition of crisis in dementia care. BMC Geriatr. 2013;13:10 10.1186/1471-2318-13-10 23374634 PMC3579755

