# Supplementary material for: The Influence of Primary Care Quality on Hospital Admissions for People with Dementia in England: A Regression Analysis
Source: PLoS One. 2015 Mar 27;10(3):e0121506. doi: 10.1371/journal.pone.0121506 (PMC4376688; doi:10.1371/journal.pone.0121506)
Supplement: S1 Appendix — (PDF) [file pone.0121506.s001.pdf]

S1 Appendix for 'Hospital admissions for dementia in England: the effect of primary care quality'

P. Kasteridis, A. Mason, M. Goddard, R. Jacobs, R. Santos, G. McGonigal

The material contained herein is supplementary to the article named in the title and submitted for consideration to PLOS ONE

Methodology for adjusting the predicted dementia registers

Prediction of QOF practice registers using published estimates of age-sex specific prevalence figures ignores that the proportion of patients in nursing homes might vary even across practices with the same age/gender composition. Because the risk of dementia for those in nursing homes is significantly higher, the predicted register should also depend on the proportion of practice patients in nursing homes. This calls for an adjustment in the calculation of predicted registers which we carry out as follows.

The risk of dementia for the population of a practice is given by

$$\Pr(D) = \Pr(D \mid NH)\Pr(NH) + \Pr(D \mid NNH)\Pr(NNH) \tag{1}$$

where $\Pr(NH)$ denotes the probability that a patient is in nursing home, $\Pr(D \mid NH)$ the probability that patients in nursing homes suffer from dementia, $\Pr(D \mid NNH)$ the probability that patients not in nurding homes have dementia, and $\Pr(NNH) = 1 - \Pr(NH)$.

For a practice with the average proportion of patients in nursing homes, the risk is given by

$$\overline{\Pr(D)} = \Pr(D \mid NH)\overline{\Pr(NH)} + \Pr(D \mid NNH)\overline{\Pr(NNH)} \tag{2}$$

where $\overline{\Pr(NH)}$, and $\overline{\Pr(NNH)}$ are probabilities averaged over all practices.

Let $\overline{p}$ be the predicted register as we calculated it using the tabulated age-gender specific

prevelance rates. It can be expressed as equation (3) where N is the practice size and $\overline{\Pr(D)}$ is the risk of dementia for the practice. Note that we are using the upper bar probability $\overline{\Pr(D)}$ because the predicted register was calculated using prevalence rates that assume the average (over all practices) proportion of patients in nursing homes.

$$\overline{p} = \overline{\Pr(D)} \times N \tag{3}$$

The adjusted predicted register is given by equation (4)

$$\widetilde{p} = [\Pr(D \mid NH)\Pr(NH) + \Pr(D \mid NNH)\Pr(NNH)] \times N \tag{4}$$

or using (3) it is written as (5)

$$\widetilde{p}=\overline{p}\frac{\Pr(D\mid NH)\Pr(NH)+\Pr(D\mid NNH)\Pr(NNH)}{\overline{\Pr(D)}} \tag{5}$$

Solving (2) with respect to $\Pr(D\mid NNH)$ we get (6)

$$\Pr(D\mid NNH)=\frac{\overline{\Pr(D)}-\Pr(D\mid NH)\overline{\Pr(NH)}}{\overline{\Pr(NNH)}} \tag{6}$$

Plugging $\Pr(D\mid NNH)$ from (6) and $\overline{\Pr(D)}$ from (3) into (5) we obtain

$$\begin{aligned}
\widetilde{p} &= \overline{p}\frac{\Pr(D\mid NH)\Pr(NH)+\frac{\frac{\overline{p}}{N}-\Pr(D|NH)\overline{\Pr(NH)}}{\overline{\Pr(NNH)}}\Pr(NNH)}{\frac{\overline{p}}{N}} \\
&= N\Pr(D\mid NH)\Pr(NH)+\frac{\overline{p}}{\overline{\Pr(NNH)}}-\frac{N\Pr(D\mid NH)\overline{\Pr(NH)}\Pr(NNH)}{\overline{\Pr(NNH)}} \\
&= N\Pr(D\mid NH)\left[\frac{\Pr(NH)\overline{\Pr(NNH)}-\overline{\Pr(NH)}\Pr(NNH)}{\overline{\Pr(NNH)}}\right]+\frac{\overline{p}}{\overline{\Pr(NNH)}}
\end{aligned} \tag{7}$$

In equation (7), the probabilities $\Pr(NH)$, $\Pr(NNH)$, $\overline{\Pr(NH)}$, $\overline{\Pr(NNH)}$ are known while $\Pr(D\mid NH)$ is estimated to 0.669 (Knapp M, Prince M, Albanese E, Banerjee S, Dhanasiri S, Fernandez J-L, et al. Dementia UK: a report into the prevalence and cost of dementia. London: Alzheimer's Society, 2007).
