# Supplementary material for: The Influence of Primary Care Quality on Hospital Admissions for People with Dementia in England: A Regression Analysis
Source: PLoS One. 2015 Mar 27;10(3):e0121506. doi: 10.1371/journal.pone.0121506 (PMC4376688; doi:10.1371/journal.pone.0121506)
Supplement: S2 Appendix — (PDF) [file pone.0121506.s002.pdf]

S2 Appendix for 'Hospital admissions for dementia in England: the effect of primary care quality'

P. Kasteridis, A. Mason, M. Goddard, R. Jacobs, R. Santos, G. McGonigal

The material contained herein is supplementary to the article named in the title and submitted for consideration to PLOS ONE.

Calculation of IRR for variables involving interaction terms

This note explains how the IRR for the Attendance Allowance (AA) and deprivation (IMD) variables reported in table 3 and 4 in the manuscript are calculated. AA is a continuous variable measuring the percentage of AA claimants and IMD_L, IMD_M, IMD_H are dummy variables indicating low, medium and high levels of deprivation:

AA: % of AA claimants
IMD_L : % of people age 60 or older living in income deprivation is <20%
IMD_M : % of people age 60 or older living in income deprivation is 20% - 35%
IMD_H : %of people age 60 or older living in income deprivation is >35%

Using interaction terms we are able to calculate incident rate ratios for AA at different deprivation levels. We denote these IRRs as:

$IRR_{AA|IMD_L}$: IRR for AA given that deprivation is low
$IRR_{AA|IMD_M}$: IRR for AA given that deprivation is medium
$IRR_{AA|IMD_H}$: IRR for AA given that deprivation is high
Similarly we denote the IRRs for medium and high deprivation as IRR_{IMD_M} and IRR_{IMD_H}.

Suppressing practice and time subscripts, the conditional mean for the Poisson model with multiplicative gamma distributed random effects is:

$$E[y|Z,\theta] = \exp\{\alpha(AA) + \beta_1(IMD_M) + \beta_2(IMD_H) + \gamma_1(AA)*(IMD_M) + \gamma_2(AA)*(IMD_H) + X\delta\} \quad (1)$$

where $Z = (AA, IMD_M, IMD_H)$ is the vector of all explanatory variables and $\theta = (\alpha, \beta_1, \beta_2, \gamma_1, \gamma_2, \delta)$ is vector of parameters.

Then the IRRs are calculated as follows :

$$IRR_{AA|IMD_L} = \frac{\exp\{\alpha(AA+1) + X\delta\}}{\exp\{\alpha(AA) + X\delta\}} = \exp(\alpha) \tag{2}$$

$$IRR_{AA|IMD_M} = \frac{\exp\{\alpha(AA+1) + \beta_1 + \gamma_1(AA+1) + X\delta\}}{\exp\{\alpha(AA) + \beta_1 + \gamma_1(AA) + X\delta\}} = \exp(\alpha + \gamma_1) \tag{3}$$

$$IRR_{AA|IMD_H} = \frac{\exp\{\alpha(AA+1) + \beta_2 + \gamma_2(AA+1) + X\delta\}}{\exp\{\alpha(AA) + \beta_2 + \gamma_2(AA) + X\delta\}} = \exp(\alpha + \gamma_2) \tag{4}$$

$$IRR_{IMD_M} = \frac{\exp\{\alpha(AA) + \beta_1(IMD_M+1) + \gamma_1(AA)(IMD_M+1) + X\delta\}}{\exp\{\alpha(AA) + \beta_1(IMD_M) + \gamma_1(AA)(IMD_M) + X\delta\}} = \exp(\beta_1 + \gamma_1 AA) \tag{5}$$

$$IRR_{IMD_H} = \frac{\exp\{\alpha(AA) + \beta_2(IMD_H+1) + \gamma_2(AA)(IMD_H+1) + X\delta\}}{\exp\{\alpha(AA) + \beta_2(IMD_H) + \gamma_2(AA)(IMD_H) + X\delta\}} = \exp(\beta_2 + \gamma_2 AA) \tag{6}$$
